# Cryo-EM samples of gas-phase purified protein assemblies using native electrospray ion-beam deposition

**DOI:** 10.1039/d2fd00065b

**Published:** 2022-09-06

**Authors:** Tim K. Esser, Jan Böhning, Paul Fremdling, Tanmay Bharat, Joseph Gault, Stephan Rauschenbach

**Affiliations:** a Department of Chemistry, University of Oxford Oxford OX1 3TF UK stephan.rauschenbach@chem.ox.ac.uk; b Sir William Dunn School of Pathology, University of Oxford South Parks Road Oxford OX1 3RE UK; c Structural Studies Division, MRC Laboratory of Molecular Biology Francis Crick Avenue Cambridge CB2 0QH UK; d Max Planck Institute for Solid State Research Heisenbergstrasse 1 Stuttgart DE-70569 Germany

## Abstract

An increasing number of studies on biomolecular function indirectly combine mass spectrometry (MS) with imaging techniques such as cryo electron microscopy (cryo-EM). This approach allows information on the homogeneity, stoichiometry, shape, and interactions of native protein complexes to be obtained, complementary to high-resolution protein structures. We have recently demonstrated TEM sample preparation *via* native electrospray ion-beam deposition (ES-IBD) as a direct link between native MS and cryo-EM. This workflow forms a potential new route to the reliable preparation of homogeneous cryo-EM samples and a better understanding of the relation between native solution-phase and native-like gas-phase structures. However, many aspects of the workflow need to be understood and optimized to obtain performance comparable to that of state-of-the-art cryo-EM. Here, we expand on the previous discussion of key factors by probing the effects of substrate type and deposition energy. We present and discuss micrographs from native ES-IBD samples with amorphous carbon, graphene, and graphene oxide, as well as landing energies in the range between 2 and 150 eV per charge.

## Abstract 2

Cryo-EM of samples prepared from mass-selected and soft-landed protein complexes creates a direct link between structure and complementary chemical information.

## Graphical abstract

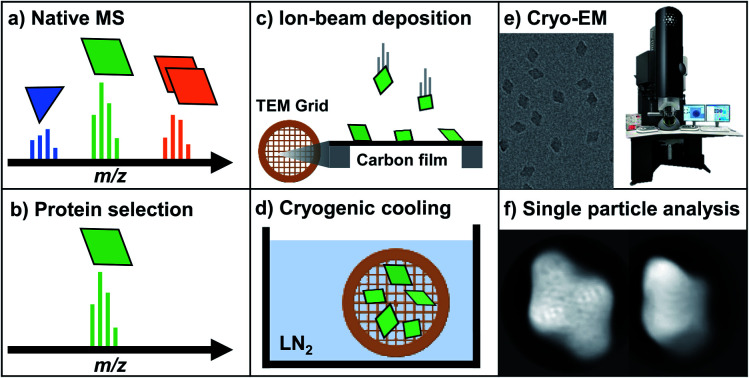

## Introduction

Understanding the function of biological macromolecules, in order to reveal causes and cures for diseases, requires detailed information on their structure (conformations, flexibility, stability), as well as their interactions (ligands, complex assemblies, native environment). Cryo electron microscopy (cryo-EM) has become the method of choice to obtain high-resolution structures of molecules that are not amenable to crystallization and are too large for NMR.

In a typical cryo-EM experiment, samples are prepared by applying solutions to TEM grids, blotting any excess with filter paper, and quickly submerging into liquid ethane to embed the biomolecules in thin layers of vitreous ice, which preserves the native environment^1^ and reduces the effects of radiation damage.^2^ In addition, molecules are imaged at a low electron dose, which typically requires hundreds of thousands of two-dimensional (2D) single particle images to be combined using single particle analysis (SPA) to reach high-resolution three-dimensional (3D) EM density maps.^3^

Despite major advances,^4,5^ sample preparation still continues to be “time-consuming and resource-intensive”, “present a major challenge” and is identified as “the main impediment of the SPA workflow”, as stated in recent reviews.^5–7^ Challenges in sample preparation include denaturation at the air–water interface, heterogeneous samples including aggregates, fragments, and contaminants, preferred particle orientation, high affinity to the support layer, as well as inhomogeneous ice-thickness.

Therefore, obtaining a high-resolution structure can still be challenging or impossible for many biomolecules. Complementary information, in particular from structurally sensitive mass spectrometry (MS) based techniques, including native MS, hydrogen deuterium exchange (HDX), ion mobility spectrometry (IMS), crosslinking mass spectrometry (XL-MS), and different forms of ion activation, can help to find optimal sample conditions, interpret and refine 3D structures, reveal native interactions, and provide information on small ligands and flexible protein regions that may have lower resolution in cryo-EM density maps, due to conformational or chemical heterogeneity.^6,8–15^

These studies indicate the potential of establishing a direct link between MS and cryo-EM, to allow for improved correlation between complementary chemical information and high-resolution structures. Electrospray ion-beam deposition (ES-IBD), also known as soft landing or preparative mass spectrometry, allows ultra-clean and molecularly pure samples to be prepared on solid surfaces for further analysis.^16–18^ This method has been used to investigate properties of molecules in the mass range from a few Daltons to a Mega-Dalton, using various substrates and imaging techniques, including SPM,^18–23^ room-temperature TEM,^24–27^ and low-energy electron holography (LEEH).^28,29^ It has been shown that viruses and enzymes can retain biologic activity after ionization, exposure to the vacuum, and deposition.^30–32^ Furthermore, in negative stain TEM of deposited protein complexes, retention of a globular shape was revealed, but higher resolution features could not be observed.^24^

We have recently developed a native mass spectrometer with ion-beam deposition capability for the preparation of mass-selective and ice-free samples for imaging under cryo conditions using native ES-IBD.^33,34^ Our setup is based on a commercial, high-resolution, tandem mass spectrometer (Thermo Scientific™ Q Exactive™ UHMR mass spectrometer), to which we have added a custom deposition stage. With this instrument, we have shown that even the non-covalently bound tetramer of alcohol dehydrogenase (ADH) preserves its enzymatic activity after deposition onto a dry carbon substrate.^34^

In native ES-IBD, protein assemblies are transferred into the gas phase in a near-native state *via* native electrospray ionization.^11^ The molecule of interest is separated from fragments, aggregates, and contaminants using mass selection, most often performed using a quadrupole mass analyzer. The mass-selected ion-beam is then deposited with defined deposition energy on TEM grids with carbon films. Grids are removed from the deposition chamber and manually plunged into liquid nitrogen, followed by cryo transfer to the microscope. Samples are then imaged at cryogenic temperature and micrographs are processed according to established SPA procedures, despite the absence of ice, resulting in 2D classes and 3D EM density maps.

Our initial results demonstrated the preparation of samples of mass-selected protein complexes with controlled particle density and retention of 2D and 3D shapes, albeit at a resolution that did not allow the determination of the secondary structure.^33^ To approach a resolution comparable to that of state-of-the-art cryo-EM, we need to understand the effects at each step of ES-IBD sample preparation on the protein structure and image quality in greater detail. The most relevant factors in need of systematic characterization and improvement include dehydration/desolvation, landing at hyperthermal kinetic energy at room temperature, molecule–substrate interaction, and sample transfer under ambient conditions.

In this study, we explore the imaging of proteins by cryo-EM following their deposition by native ES-IBD. We show that we can measure the shape of a range of protein assemblies in a mass range from 150 to 800 kDa, and demonstrate the effects of different substrates and deposition energies.

## Experimental methods

### Native electrospray ion-beam deposition (ES-IBD)

**Fig. 1:**
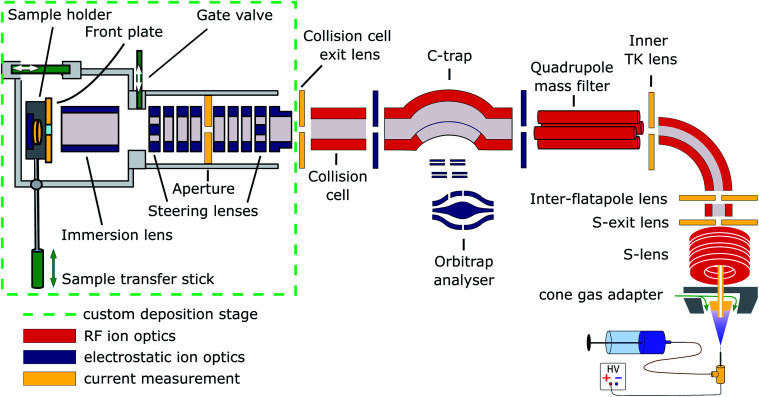
Schematic of the native ES-IBD instrument, consisting of a commercial, high-resolution mass spectrometry platform (Thermo Scientific™ Q Exactive™ UHMR mass spectrometer, right side) and home-made landing stage and sample holder (left side). The sample holder contains two sample slots and a retarding grid energy detector. A custom software is used to steer and focus the ion beam onto the detector or samples and monitor coverage during deposition. The instrument combines RF (red) and DC (blue) ion optics. DC ion optics color-coded in yellow are connected to picoammeters, to allow for current measurement to optimize ion-beam transmission. The aperture of the S-exit lens was increased from 1.4 to 2.5 mm, increasing the transmission of native proteins up to tenfold. Figure reproduced from Fremdling *et al.*^34^

Cryo-EM samples were prepared mass-selectively using native electrospray ion-beam deposition (native ES-IBD) on a customized deposition instrument based on a Q-Exactive™ UHMR mass spectrometer, shown in Fig. 1 and described in detail elsewhere.^33,34^ Protein solutions were prepared using a standard native MS workflow described below. Protein assemblies were transferred into the gas phase *via* native electrospray ionization using gold coated borosilicate capillaries, prepared in house. The complex of interest was separated from fragments, aggregates, and contaminants using mass-selection in the quadrupole. The instrument mode was then switched to guide the mass-selected ion beam through the collision cell, where it was thermalized, before entering a custom deposition stage and reaching a custom, room-temperature sample holder at a pressure of 10^−6^ mbar.

The sample holder comprises a retarding-grid ion-current detector, which records the ion-beam intensity, total beam energy, and beam-energy distribution, as well as two sample positions with variable DC potential, which allows the deposition energy to be controlled and to monitor the total charge deposited. After measurement of the beam energy, the deposition energy was defined to typically 2 eV per charge. The ion-beam was then deposited on TEM grids with amorphous carbon, graphene, or graphene-oxide films, until the desired coverage was reached. A density of more than 1000 particles per μm^2^ in the grid center was typically achieved within 30 minutes, due to a ten-fold increase of transmission after modification of the ion source. Grids were removed from the deposition chamber using a load-lock, transferred under ambient conditions, and manually plunged into liquid nitrogen, followed by cryo transfer to the microscope. Samples were then imaged and micrographs were processed according to established single-particle analysis (SPA) procedures.

### Sample preparation and native mass spectrometry

Soluble proteins, alcohol dehydrogenase (ADH, A7011-15KU), β-galactosidase (β-gal, G3153-5MG), ferritin (F4503-25MG), and GroEL (chaperonin 60, C7688-1MG), were purchased from Sigma Aldrich and used without further purification unless otherwise specified. Ammonium acetate (7.5 M, A2706-100ML), MeOH (1060352500), acetone (1000145000) and buffer components for the reconstitution of GroEL, Tris (93362-250G), KCl (P9541-500G), EDTA (DS-100G), MgCl_2_ (63068-250G) and ATP (A6419-1G), were also purchased from Sigma Aldrich. All concentrations were calculated with respect to the most abundant oligomers. Lyophilized powders of ADH and β-gal were resuspended in 200 mM ammonium acetate (pH 6.9) to a final concentration of 50 μM. The saline ferritin stock solution had a concentration of 260 μM. GroEL was reconstituted from lyophilized powder as described previously.^33^

**Fig. 2:**
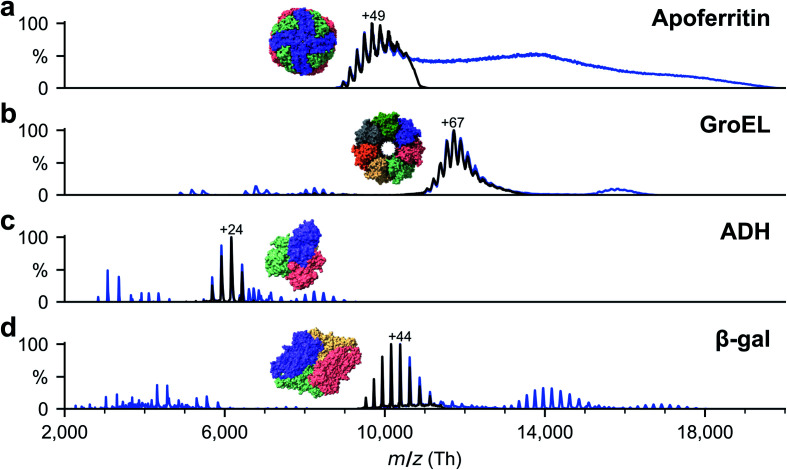
Non-activating, native mass spectra of apo/holo-ferritin, GroEL, ADH, and β-gal. For native ES-IBD experiments, native oligomers were mass selected (black lines), *i.e.*, tetramer for ADH and β-gal, tetradecamer for GroEL and 24-mer for apoferritin. Full spectra (blue lines) include non specific aggregates, fragments, and contaminants. Holoferritin (right of panel a) shows no resolved charge states due to the wide size distribution of iron cores in our sample. For all other proteins, the most abundant charge states are labeled. They are much lower than typical charge states from denatured samples, indicating folded gas-phase structures.

All proteins were desalted by passing through two P6 buffer exchange columns (7326222, Biorad) and equilibrated with 200 mM ammonium acetate (pH 6.9). If applicable, they were then diluted in 200 mM ammonium acetate (pH 6.9) to reach the concentration used for native MS: 5 μM (ADH), 10 μM (β-gal), 8 μM (ferritin) and 5 μM (GroEL). Buffer exchange was always done on the day of deposition. General instrument conditions were as follows: source DC offset = 21 V, S-lens RF level = 200 (300 Vp–p), transfer capillary temperature = 60 °C, ion transmission settings set to “high *m*/*z*” (700 Vp–p for the injection flatapole, and 900 Vp–p for the bent flatapole, transfer multipole, and collision cell), detector optimization “high *m*/*z*”, injection flatapole = 5 V, interflatapole lens = 4 V, bent flatapole = 2 V, transfer multipole = 0 V, collision-cell pressure setting 7 (N_2_), collision-cell multipole DC −5 V and collision-cell exit-lens −15 V. Fig. 2 shows the corresponding mass spectra. The full spectra (blue lines) include non specific aggregates, fragments, and contaminants in addition to the peaks corresponding to the intact native species. For sample preparation, we selected the native oligomers using the quadrupole mass filter (black lines).

### TEM grid preparation

Copper TEM grids with a mesh size of 400 were purchased from Agar Scientific, including 3 nm amorphous carbon on lacey carbon (AGS187-4), monolayer graphene on holey carbon (AGS179-GO4), and plain holey carbon (AGS174-3). A graphene oxide layer was added to the latter by plasma-cleaning for five minutes, drop casting a 3 μL graphene oxide suspension (763705-25ML, Sigma Aldrich), diluted in water to 0.2 mg mL^−1^, blotting with filter paper (11547873, Fisherbrand) after one minute, followed by three washing and blotting steps with water. No further treatment was applied to the grids before deposition.

### Image acquisition and processing

All micrographs were collected using microscopes at the COSMIC Cryo-EM facility at South Parks Road, University of Oxford, UK. The micrograph of apo/holo-ferritin on graphene oxide was acquired on a Talos F200C (Thermo Fisher Scientific) cryo-TEM equipped with a Ceta 16M CMOS camera. All other data was acquired on a Talos Arctica 200 kV cryo-TEM with a Falcon 4 direct electron detector (both Thermo Fisher Scientific). Manual and automated data acquisition were controlled using EPU software (Thermo Fisher Scientific).

**Fig. 3:**
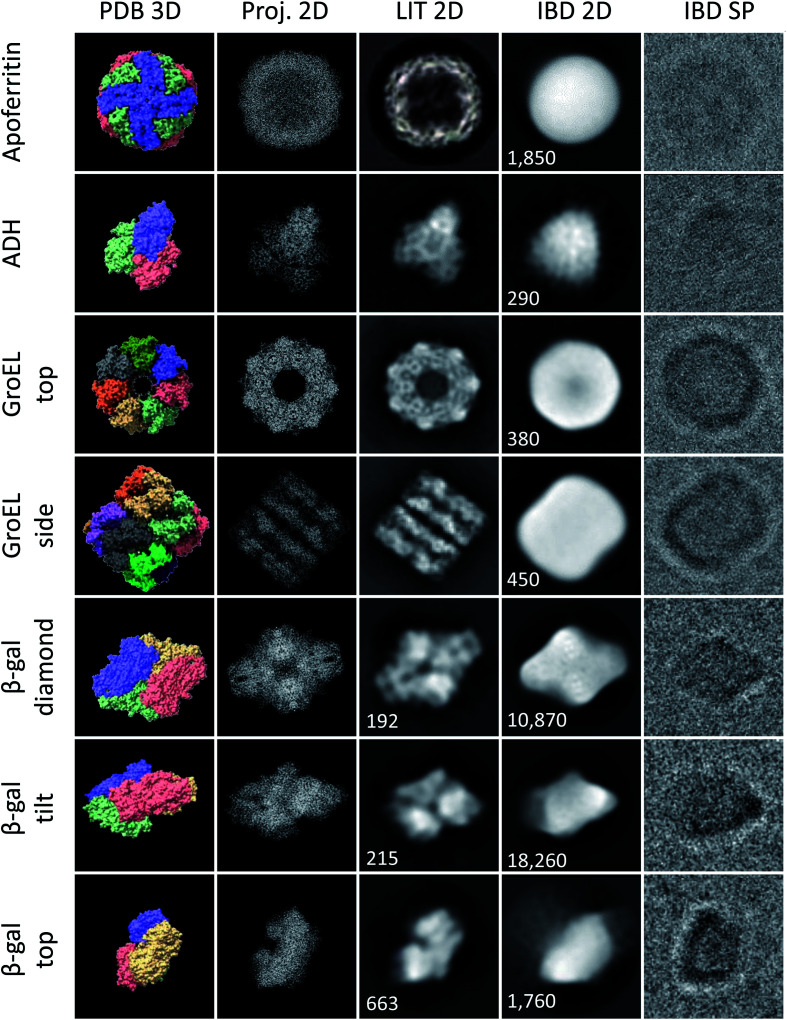
Characteristic shapes from ice-free native ES-IBD samples of apoferritin, GroEL, ADH, and β-gal. Panels show, from left to right, 3D models from the PDB (PDB 3D), corresponding 2D projections (Proj. 2D), 2D classes from plunge-frozen cryo-EM samples from the literature (LIT 2D), and 2D classes (IBD 2D) and representative single particles (IBD SP) from ice-free native ES-IBD samples. 3D PDB models were rendered with ChimeraX^39^ using PDB entries 7A6A (apoferritin), 5W0S (GroEL), 7KCQ (ADH), and 6CVM (β-gal). 2D classes from the literature were taken from Yip *et al.*^40^ (apoferritin), Roh *et al.*^41^ (GroEL), Guntupalli *et al.*^42^ (ADH), and the RELION 3.0 tutorial data set (β-gal). The number of particles in the 2D classes is given where available. Figure adopted in part from Esser *et al.*^33^

Typically, 50 micrographs were recorded per sample, giving up to 3000 particles. For β-gal, a larger data set with 50 000 particles was recorded. A range of defocus settings between −1 and −3 μm was used. Magnification was at least 90 000, corresponding to a pixel size smaller or equal to 1.52 Å. In general, micrographs were recorded in MRC format at an exposure of 40 *e* Å^−2^. For β-gal, a larger data set of 3000 EER^35^ movies was collected, providing 50 000 particles. The magnification for this collection was 240 000, corresponding to a pixel size of 0.59 Å. The total exposure was 40 *e* Å^−2^, but the 2D classes shown in Fig. 3 were obtained using only the first 5 *e* Å^−2^. For the unfiltered micrographs shown in this work, the color range was adjusted to the data range, but no data was cut off and no non-linear adjustments were applied.

For SPA, data was processed using RELION 3.1.^36^ Motion-corrected MRC files were generated from EER files, using RELION’s own implementation of the MotionCor2 algorithm.^37^ Contrast transfer functions (CTFs) were estimated using CTFFIND 4.1 (ref. 38) and used for CTF correction in the following steps. The high contrast obtained for native ES-IBD samples allowed for reliable automated particle picking based on a Laplacian-of-Gaussian (LoG) filter. Particles were extracted in approx. 300 Å × 300 Å boxes and scaled down only for the EER data set by a factor of 2. An initial 2D classification step was used to remove incorrectly picked particles and subsequent 2D classification produced the 2D classes shown in here.

## Results and discussion

We have applied native ES-IBD and cryo-EM to image folded and assembled protein complexes, including apoferritin, alcohol dehydrogenase (ADH), β-galactosidase (β-gal), and GroEL, after gas-phase purification by mass selection followed by a gentle surface deposition. In Fig. 3, we compare single particles (IBD SP) and 2D classes (IBD 2D) for several characteristic projection angles to 3D models from the PDB (PDB 3D), corresponding 2D projections (Proj. 2D), and 2D classes from the literature (LIT 2D).

The micrographs show a very high contrast, allowing single particles to be identified, including shape and orientation, without class averaging or image manipulation. For all systems, the direct comparison to projections demonstrates the conservation of the overall protein shape. Beyond this agreement, some of the proteins show characteristic features, such as the heptameric symmetry and the cavity for GroEL.

However, to date, lower resolution is observed compared to samples with vitreous ice, which means that the secondary structure within the protein shape cannot be identified. Removed from the liquid environment, the protein dehydrates and interacts with the background gas in the mass spectrometer, as well as with the TEM grid surface upon impact and as an adsorbate. These interactions differ from those in the native environment, and can locally alter the structure. If this structural change is partially random, it can lead to the lack of detail observed in 2D classes obtained after averaging in SPA.

Fig. 3 demonstrates the current performance and limitations of the native ES-IBD workflow in its application for cryo-EM sample preparation. Clearly, an improved sample quality is needed for applications beyond determining the general shape of proteins. For that, it is essential to understand the interactions, which can lead to the random deformation of the protein. In the following, we present observations of the protein–substrate interaction and deposition energy as observed by cryo-EM imaging after native ES-IBD.

### Substrate

**Fig. 4:**
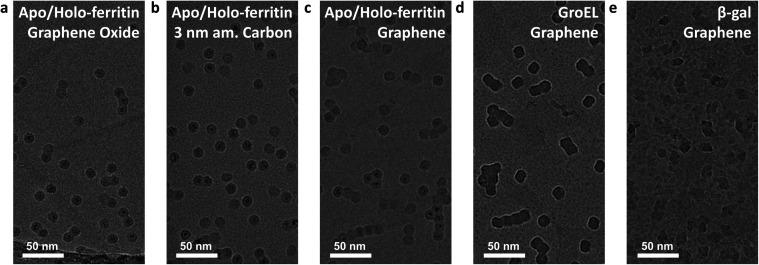
Deposition and imaging on different substrates. (a–c) Holo/apo-ferritin on different substrates: (a) graphene oxide, (b) 3 nm amorphous carbon, (c) freestanding single layer graphene. (d) GroEL and (e) β-gal deposited on freestanding single layer graphene. All depositions were performed with a deposition energy of less than 5 eV per charge. The contrast on all substrates is comparable, but at room temperature, only amorphous carbon reduces the thermal motion sufficiently to avoid aggregation. Here, grid squares with comparable particle density were chosen. In general, the entire grid is covered by particles and particle density increases continuously towards the grid center.

All native ES-IBD data presented in Fig. 3 were obtained using grids with 3 nm amorphous carbon membranes. Fig. 4 shows a comparison of different substrates. We repeated imaging of GroEL, apo/holo-ferritin and β-gal using grids with a graphene film. For apo-/holoferritin, we also show samples with graphene oxide (GO) and 3 nm amorphous carbon.

Overall, the resulting images are of comparable quality and high contrast. The most notable difference is the particle distribution. While particles are randomly distributed on amorphous carbon, they frequently occur as aggregates on graphene oxide and graphene. On graphene oxide, an increasing particle density towards the edge of the foil holes is observed, likely caused by an increasing film thickness and number of defects. On graphene, chain-like aggregates are common, consistent with survey images of proteins on graphene obtained by LEEH.^28^

This observation suggests that particles are less strongly bound on graphene and thin graphene oxide and are able to undergo thermally activated motion after deposition and adsorption. It is well known that an organic film forms on clean graphene after several hours under ambient conditions.^43,44^ As the grids were never exposed to any liquids, we assume that this organic film is causing the heterogeneous contrast between the protein particles on graphene, in particular in Fig. 4e. This renders the intrinsically hydrophilic graphene hydrophobic. In solution-based sample preparation, plasma cleaning is used to obtain a clean hydrophilic substrate that allows for even wetting. In contrast, in a solution-free sample preparation, the organic film or other surface modifications could be used to reduce thermal diffusion and, thus, promote the imaging of individual, non-aggregated particles.

The differences in particle mobility suggest varying interaction strength for the substrates used. However, at least for the substrates tested here, the effect of interaction with the substrate is not limiting the image quality. For all tested substrates, we observe a strong preferred orientation. Given the quality of 2D classes, this is currently not limiting either. If the sample quality can be significantly improved, preferred orientation will need to be addressed, either by surface modification or the use of cryo electron tomography and subtomogram averaging.

### Deposition energy

In the soft-landing regime, the deposition energy is typically 2 eV per charge or lower, corresponding to a total kinetic energy below 100 eV for a large protein complex with a charge state below +50, such as β-gal. This is a significant amount of energy, much higher than in a thermal collision, and it is sufficient to break several covalent bonds or deform the protein if the energy is localized and cannot be dissipated sufficiently fast.

**Fig. 5:**
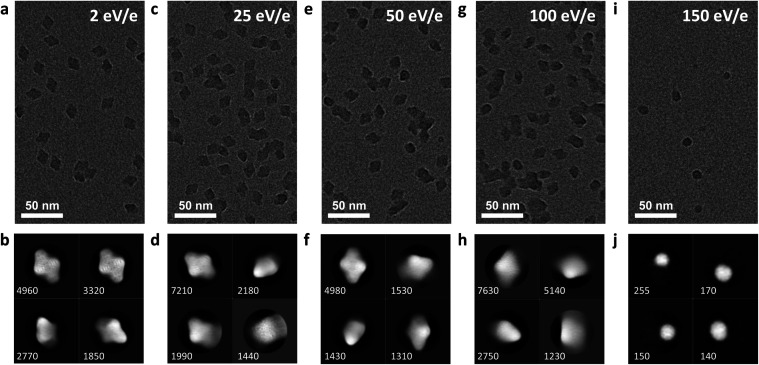
Micrographs of five samples, prepared using at a mean deposition energy of 2, 25, 50, 100 and to 150 eV per charge, are shown in panels (a, c, e, g, and i). All other parameters, including settings of the mass spectrometer, microscope, and data analysis, were kept identical. Panels (b, d, f, h, and j) show corresponding 2D classes. With increasing deposition energy, internal features and the overall shape become more diffuse. At a deposition energy of 150 eV per charge, fragments appear instead of fully assembled tetramers.

We investigated the effect of the deposition energies in the range from 2 to 150 eV per charge on the structure of β-gal. The corresponding micrographs and 2D classes are shown in Fig. 5. This protein complex is a tetramer with a mass of 466 kDa and a charge state of +45. In soft landing, we used a deposition energy of 2 eV per charge. The particle will have a total kinetic energy of 90 eV and a speed of 190 m s^−1^ when landing. The time required to move its own height (≤17 nm) at that speed, 90 ps, is an upper limit for the duration of the landing event.

Under these conditions, 2D classes of β-gal show a characteristic substructure, distinctly different from that obtained from samples with vitrified ice. The substructure disappears at higher deposition energies, indicating that it is a real feature of our sample, instead of an artifact from imaging or the classification algorithm. The overall shape remains the same, even at a deposition energy of 100 eV per charge.

The total energy distribution of the ion beam has a full width at half maximum of 1 eV per charge.^34^ Thus, most proteins land with an energy in the range from 1.5 to 2.5 eV per charge. We do not see any distribution in particle quality corresponding to this deposition energy range, but cannot exclude that even lower deposition energies are required to increase sample quality. Alternatively, improved energy dissipation upon impact could be achieved using an inert-gas buffer layer on the surface.^45^

At a deposition energy of 150 eV per charge, we see a distinct change in the micrograph and 2D classes. Instead of the shape of assembled tetramers, we see smaller, round features. Particles land with a total kinetic energy of 6.75 keV, corresponding to a speed of 1770 m s^−1^. At this speed, the upper estimate for the duration of the landing event is only 10 ps. At this short time scale, the energy imparted from the collision, typically into the softest vibrational mode, cannot be effectively distributed over the molecule before non-covalent bonds are broken by large oscillation amplitudes. For collisions of smaller molecules with surfaces at hyperthermal energies, similar reaction mechanisms have recently been shown to break covalent and non-covalent bonds.^46,47^

Hence, we assign the features in Fig. 5i to subunits of β-gal, which are formed as a consequence of dissociation, caused by the collision. Here, we have essentially performed a surface induced dissociation (SID) experiment. However, we observe fragments that remained on the surface, instead of fragment ions that are scattered back into the gas phase, as in conventional SID.^48^ We cannot currently determine if all or just a fraction of fragments created in our experiment adsorb onto the surface. In principle, this is possible by determining the total number of fragments on the grid and comparing it to the number of deposited protein complexes.

If the resolution can be improved, this variant of SID has the potential to analyze the 3D structure of the fragments, in addition to binding sites, dissociation or fragmentation thresholds, and local mechanical properties. As the structure of fragments after dissociation may be highly heterogeneous, a true single particle technique like LEEH may be needed to take full advantage of this approach.^29^ Furthermore, the deformation of the protein assembly observed at 100 eV per charge, below the dissociation threshold, contains information not available in SID experiments. This structural change is indicative of the mechanical properties of the protein.

## Conclusions

We have demonstrated the ability to obtain protein shapes by cryo-EM after gas-phase purification, and analyzed the effect of various substrates and deposition energies on native ES-IBD samples. Altering the substrate and deposition energy had no immediate dramatic effect on the observed protein shapes, unless very high deposition energies were used. While this does not exclude these factors from being responsible for the reduced spatial resolution in cryo-EM single particle analysis, it suggests that other factors are currently limiting.

A simultaneously developed workflow, based on matrix landing and negative staining, has demonstrated the potential of a rehydration step, for example by landing directly into liquid glycerol.^27^ Those experiments suggests that the effect of electrospray and temporary exposure to the vacuum are small or reversible. Indeed, it has been shown that proteins can assume kinetically trapped gas-phase structures, but the native secondary structure is typically largely preserved and gas-phase proteins are able to refold, at least partially, upon rehydration.^49–51^ Currently, the matrix landing approach cannot provide access to high-resolution internal structure, due to the use of negative stain.

While it is desirable to connect this and other approaches that include a controlled rehydration step to cryo-EM, gas-phase protein structures are interesting in their own right. After all, mass spectrometry and the broad range of methods built on it are based on the premise that a near-native protein structure is retained in the gas-phase. Direct imaging after transfer through the gas-phase and various forms of gas-phase activation, such as SID, may reveal a more detailed picture of the nature of structural change than, for example, rotationally averaged collisional cross sections, obtained through IMS. This may provide important information to help interpret and validate the results of other mass spectrometry-based techniques.

Another key parameter of the native ES-IBD workflow that remains to be characterized is the substrate temperature. Deposition onto cryogenically-cooled grids could reduce thermal diffusion in a controlled way. This would enable the use of clean graphene films to obtain the best possible contrast, and likely reduce the thermally activated structural changes during and after deposition. To avoid contamination, cold samples will need to be prepared in ultra-high vacuum (UHV) and transferred under UHV or in liquid nitrogen. To this end, we are currently implementing a cryo, *in vacuo* sample holder. We are confident that higher resolution, and thus new applications, will become possible after further optimization of the native ES-IBD workflow.

## Conflicts of interest

There are no conflicts of interest to declare.
